# Untargeted and Oxylipin-Targeted Metabolomics Study on the Plasma Samples of Primary Open-Angle Glaucoma Patients

**DOI:** 10.3390/biom14030307

**Published:** 2024-03-05

**Authors:** Jianming Xu, Changzhen Fu, Yaru Sun, Xin Wen, Chong-Bo Chen, Chukai Huang, Tsz Kin Ng, Qingping Liu, Mingzhi Zhang

**Affiliations:** 1 Joint Shantou International Eye Center of Shantou University and The Chinese University of Hong Kong, Shantou 515041, China; xjm@jsiec.org (J.X.); fcz@jsiec.org (C.F.); syr@jsiec.org (Y.S.); wenxin@jsiec.org (X.W.); hck@jsiec.org (C.H.); wjz@jsiec.org (T.K.N.); 2 Shantou University Medical College, Shantou 515041, China; 3 Department of Ophthalmology and Visual Sciences, The Chinese University of Hong Kong, Hong Kong 999077, China; 4 Guangdong-Hong Kong-Macao Joint Laboratory for Precision Prevention and Research of Eye Diseases, Shantou 515041, China; 5 Guangdong Provincial Engineering Technology Research Center for Precision Diagnosis and Treatment of Eye Diseases, Shantou 515041, China

**Keywords:** primary open-angle glaucoma, metabolomics, oxylipins, arachidonic acid, inflammations

## Abstract

**Purpose**: to determine the metabolomics profiles in the plasma samples of primary open-angle glaucoma (POAG) patients. **Methods**: The plasma samples from 20 POAG patients under intraocular pressure (IOP)-lowering medication treatment and 20 control subjects were subjected to the untargeted metabolomics analysis, among which 10 POAG patients and 10 control subjects were further subjected to the oxylipin-targeted metabolomics analysis by liquid chromatography–mass spectrometry analysis. The prediction accuracy of the differentially abundant metabolites was assessed by the receiver operating characteristic curves. Pathway analysis and correlation analysis on the differentially abundant metabolites and clinical and biochemical parameters were also conducted. **Results**: Untargeted metabolomics profiling identified 33 differentially abundant metabolites in the POAG patients, in which the metabolism of linoleic acid, α-linolenic acid, phenylalanine, and tricarboxylic acid cycle were enriched. The correlation analysis indicated that the differentially abundant metabolites were associated with central corneal thickness, peripapillary retinal nerve fiber layer thickness, visual field defects, and lymphocytes. The oxylipin-targeted metabolomics analysis identified 15-keto-Prostaglandin F2 alpha, 13,14-Dihydro-15-keto-prostaglandin D2, 11-Dehydro-thromboxane B2, 8,9-Epoxyeicosatrienoic acid, and arachidonic acid to be significantly decreased in the POAG patients and enriched in the arachidonic acid (AA) pathway. **Conclusions**: This study revealed that the metabolites in the arachidonic acid metabolism pathway are differentially abundant, suggesting high IOP may not be the only detrimental factor for optic nerve cell damage in this group of POAG patients. Lipid metabolism instability-mediated alterations in oxylipins and AA pathways may be important in POAG, suggesting that oxidative stress and immune-related inflammation could be valuable directions for future therapeutic strategies.

## 1. Introduction

Glaucoma is a leading cause of irreversible blindness and visual impairment predicted to affect 111.8 million people worldwide by 2040 [1,2,3]. Primary open-angle glaucoma (POAG) is the most common type of glaucoma, characterized by the progressive degeneration of retinal ganglion cells (RGCs). The pathogenesis of POAG is complex, and intraocular pressure (IOP) elevation is a reversible risk factor [1]. Clinically, the visual field defects still progress in some POAG patients even if the IOP has been lowered to the “normal range” after medication or surgical treatment. In a population-based study [4], persistent thinning in the retinal nerve fiber layer (RNFL) was found in 33% of glaucoma patients with IOP <18 mmHg and 9% of patients had progression even when the IOP is controlled at <15 mmHg. Therefore, IOP elevation may not be the only risk factor for POAG. There could be other factors contributing to progressive RGC loss in POAG.

Metabolomics offers an experimental approach to identifying differentially abundant metabolites that can assist in disease diagnosis and provide new perspectives for the disease pathogenesis [5]. Previous metabolomics studies have identified a number of differentially abundant metabolites, including amino acids [6,7,8,9,10,11,12], lipids [6,12,13,14,15,16], nucleotides [6,8,9,10], vitamins [6,13,14,15,16,17], and amides [12,13] in aqueous humor, serum, and plasma samples of POAG patients. The identified differentially abundant metabolites are enriched in amino acid metabolism, lipids metabolism, mitochondrial oxidation, senescence, biotin biosynthesis, and purine nucleotide metabolism [18,19].

Metabolites mainly participate in the progression of glaucoma by affecting mitochondrial energy metabolism, oxidative stress, and immune-inflammatory processes. Currently, metabolomic studies on POAG are limited, necessitating a comprehensive exploration of POAG’s metabolic characteristics via multi-omics approaches to provide new strategies for the diagnosis and treatment of glaucoma.

Here, this study aimed to determine the metabolomics profiles of the plasma samples from the POAG patients under IOP-lowering medication treatment. The metabolic pathways and the correlation with clinical and biochemical parameters were also evaluated.

## 2. Materials and Methods

### 2.1. Study Subjects

In total, 20 POAG patients undergoing IOP-lowering medication treatment and 20 control subjects were enrolled for the untargeted metabolomics analysis, among which 10 POAG and 10 control subjects were randomly selected for further oxylipin-targeted metabolomics analysis. The sample size was based on previous metabolomics studies [9]. This study was approved by the Ethics Committee for Human Medical Research at the Joint Shantou International Eye Center of Shantou University and The Chinese University of Hong Kong, which is in accordance with the tenets of the Declaration of Helsinki. Written informed consent was obtained from all study subjects after explaining the nature and possible consequences of the study. All of the subjects were from the same ethnic background and geographical region.

The inclusion criteria of the POAG subjects included (1) IOP >21 mmHg at diagnosis and IOP ≤21 mmHg under IOP lowering medication treatment; (2) open anterior chamber angle by gonioscopy; (3) cup-to-disk ratio (C/D) >0.5 or binocular C/D differences >0.2; (4) RNFL thinning confirmed by optical coherence tomography (OCT) examination; and (5) visual field (VF) defects confirmed by HUMPHREY visual field analyzer. Patients with secondary glaucoma, previous glaucoma surgery, ocular trauma, diabetes, and hypertension were excluded from this study. The age and sex-matched healthy control subjects had no glaucoma and other eye diseases except mild senile cataract or refractive errors and IOP ≤21 mmHg. Participants in the control group were excluded if they had hypertension, diabetes, or a history of long-term or recent medication use.

The demographic data, including age, sex, blood pressure (systolic/diastolic blood pressure), body mass index (BMI), and smoking and medication history, such as glaucoma and statin medication, were also collected (Table 1).

### 2.2. Ophthalmic Examinations and Blood Tests

All study subjects received complete ophthalmic examinations, including refraction, tonometry, slit-lamp biomicroscopy, gonioscopy, ocular biometry, visual field, OCT, and best-corrected visual acuity (logMAR) analyses. Goldmann applanation tonometry (GAT) (Haag-Streit, Konig, Switzerland) was used to measure the IOP, and slit-lamp biomicroscopy (Haag-Streit model BQ-159 900; Haag-Streit) was used to examine the anterior chamber and the lens. Non-contact partial coherence interferometry (IOL Master V3.01, 164 Carl Zeiss Meditec AG, Jena, Germany) was used to measure the ocular biometric parameters, including axial length (AL), central corneal thickness (CCT), and anterior chamber depth (ACD). Visual field was measured by the Humphrey MATRIX (Carl Zeiss, Berlin, Germany) and RNFL thickness was measured by Cirrus HD-OCT 4000 (Carl Zeiss, Berlin, Germany). Fasting peripheral blood was collected for routine blood and biochemical tests.

### 2.3. Metabolomics Analysis

Fasting peripheral blood was collected in 5 mL EDTA tubes. The tubes were immediately transferred onto ice and centrifuged at 3000 rpm for 20 min at 4 °C. The supernatant plasma was collected and stored at −80 °C before the metabolomics analysis.

The processes for the preparation of samples, the analysis of extracts, the identification of metabolites, and the quantification were carried out in Sensichip Biotechnology Co., Ltd. (Shanghai, China), following their standard procedures—the metabolomics analysis performed on LC–MS (Thermo, Ultimate 3000LC, Q Exactive) platform. The ACQUITY UPLC HSS T3 column (100 mm × 2.1 mm, 1.8 μm) (Waters, Milford, MA, USA) was utilized for the chromatographic separation, employing a binary solvent system (solvent A: 0.05% formic acid in water; solvent B: acetonitrile). The gradient elution procedures were 0–1 min, 95% A; 1–12 min, 95% A; 12–13.5 min, 5% A; 13.5–13.6 min, 95% A; and 13.6–16 min, held at 95% A. The column temperature was kept at 40 °C, with a flow rate of 0.3 mL/min and an injection volume of 5 μL. The full-scan mode (m/z ranges 70–1050) with data-dependent secondary mass spectrometry scanning (TopN = 10) was utilized. Mass spectrometry was operated in both positive and negative ion modes. The mass spectrometry parameters included heater temperature 300 °C (+) and 300 °C (−); sheath gas flow rate 45 arb (+) and 45 arb (−); aux gas flow rate 15 arb (+) and 15 arb (−); sweep gas flow rate 1 arb (+) and 1 arb (−); spray voltage 3000 V (+) and 3200 V (−); capillary temperature 350 °C (+) and 350 °C (−); and S-Lens RF level 30% (+) and 60% (−). The compounds were identified by comparing their retention time with authentic standards, accurate mass, and fragmentation patterns based on the database (http://metlin.scripps.edu, accessed on 29 September 2022).

The software SIMCA-P (V14.1, Sartorius Stedim Data Analytics AB, Umea, Sweden) was used to conduct the metabolomics analyses. To determine the differentially abundant metabolites between the POAG patients and control subjects, the principal component analysis (PCA) and orthogonal partial least squares discriminant analysis (OPLS-DA) were applied. In the OPLS-DA permutation test, the values of R^2^ and Q^2^ indicated the explainability and predictability of the model, respectively. Multiple testing correction (Benjamini–Hochberg FDR, taking FDR <0.05 as a threshold) was conducted to acquire the adjusted *p*-value. The metabolites with variable importance in projection (VIP) exceeding ± 1 and *p* < 0.05 were considered the differentially abundant metabolites. The ggplot package (v.3.3.0) was utilized to generate the hierarchical clustering map and the correlation heatmap. Furthermore, KEGG (http://www.genome.jp/kegg/, accessed on 29 September 2022) and MetaboAnalyst (http://www.metaboanalyst.ca/, accessed on 29 September 2022) were used for the pathway analysis.

### 2.4. Statistical Analysis

Independent *t*-test was used to analyze variables with normal distribution, while non-parametric Mann–Whitney U test was used to analyze variables without normal distribution. Categorical data was analyzed by Fisher’s exact test. Pearson correlation was performed between the clinical and biochemical parameters and DEMs. Receiver operating characteristic curves (ROC) were calculated to assess the prediction accuracy of the DEMs. All statistical tests were conducted using IBM SPSS STATISTICS 26 (SPSS Inc., Chicago, IL, USA). *p* < 0.05 was considered statistically significant.

## 3. Results

### 3.1. Demographics of the Study Subjects

There were no statistically significant differences in age, sex, IOP, and other ocular biometric parameters between the POAG and healthy control groups (Table 2). C/D was significantly higher in the POAG patients than the healthy control subjects (*p* < 0.001). For the blood and biochemical tests, total cholesterol (TC; *p* = 0.031) and mononuclear cells (*p* = 0.017) were significantly higher in the POAG patients than the control subjects.

### 3.2. Untargeted Metabolomics Analysis (UTMs)

**Figure 1:**
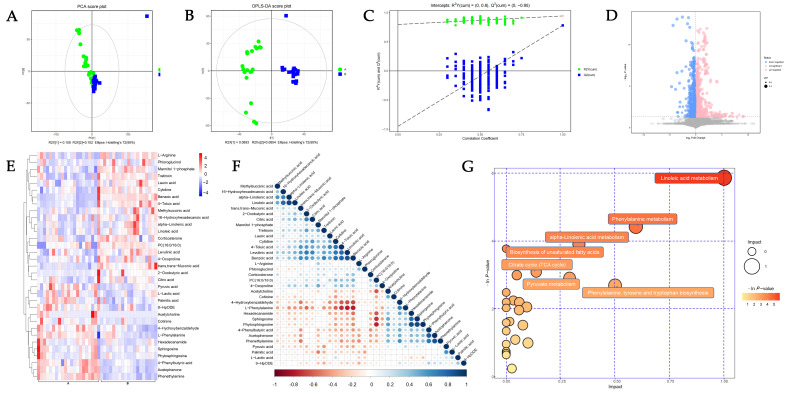
The untargeted metabolomics analysis (UTMs). (**A**) Principal component analysis (PCA) plots and (**B**) orthogonal projections to latent structures–discriminate analysis (OPLS-DA) score plots illustrate the clustering and dispersion of the two groups. (**C**) Permutation tests of OPLS-DA mode of metabolomics analysis. (**D**) The volcano plot represents the screening of differentially abundant metabolites, and the point size represents the variable importance projection (VIP) value of the OPLS-DA model. Upregulated metabolites are shown in red, downregulated metabolites in blue, and non-significantly different metabolites in gray. The hierarchical cluster analysis heatmap (**E**) of DEMs demonstrated in POAG and control group. A represents POAG, and B represents the control group. Red represents upregulation, while blue represents downregulation. Each column represents an individual sample, and each row represents differentially abundant metabolites. (**F**) The correlation between differentially abundant metabolites is shown in a correlation heatmap. Red represents a negative correlation, while blue represents a positive correlation. The size of the circle represents the Pearson coefficient. (**G**) The bubble plots indicate enrichment pathways in the plasma between POAG and control.

**Figure 2:**
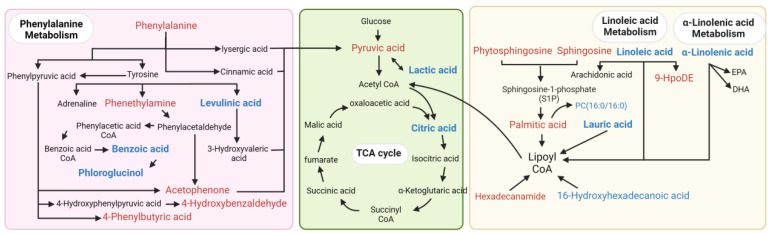
The pathway mapping of the differentially abundant metabolites. The pink area represents phenylalanine metabolism, the green area represents energy metabolism, and the yellow area represents lipid metabolism. Red color indicates the upregulation of DEMs, blue color indicates the downregulation of DEMs, and black metabolites are other important metabolites involved in the pathway.

The PCA (Figure 1A) and OPLS-DA (Figure 1B) revealed the clustering of the samples within the same group and the differences between groups. The OPLS-DA permutation test (R^2^ = 0.944, Q^2^ = 0.782) confirmed that the model was of acceptable explainability and predictability. (Figure 1C) The volcano plot identified 33 differentially abundant metabolites, of which 14 showed more abundance, and 19 were less abundance in the POAG patients (Figure 1D and Table 3), and were categorized as lipids, amino acids and their derivatives, organic acids, ketones, alkaloids, sugars, nucleotides, and neurotransmitters. Overall, 11 of the 33 differentially abundant metabolites were lipids, including polyunsaturated fatty acids, saturated fatty acids, phospholipids, sphingomyelin, steroids, and lipid peroxides. To assess the diagnostic potentials of the differentially abundant metabolites, ROC analysis was performed. The distinguished expression pattern of the 33 DEMs between POAG and the control group was visually demonstrated in the hierarchical cluster analysis heatmap (Figure 1E). The synergy and antagonism between DEMs are shown in the correlation heatmap (Figure 1F). The enrichment pathway analysis was carried out to visualize the metabolic pathways affected in POAG groups (Figure 1G). The pathway enrichment analysis demonstrated that the linoleic acid (LA) metabolism, phenylalanine metabolism, α-linolenic acid (ALA) metabolism, and tricarboxylic acid (TCA) cycle were filtered out as the enriched pathways (*p* < 0.05, impact value > 0.1) (Table 4) (Figure 2).

**Figure 3:**
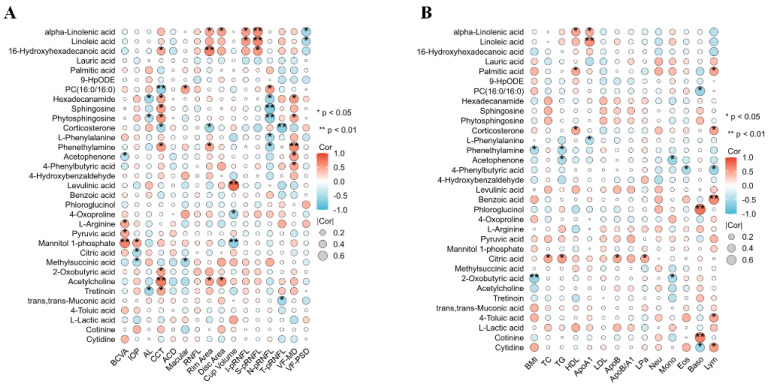
The correlation analysis of differentially abundant metabolites with the clinical and biochemical parameters. Correlation analysis between 33 DEMs and clinical (**A**) and biochemical (**B**) parameters. Clinical parameters: BCVA, IOP, AL, CCT, ACD, macular thickness, RNFL thickness, rim area, disc area, cup volume, inferior pRNFL thickness, superior pRNFL thickness, nasal pRNFL thickness, temporal pRNFL thickness, visual field mean defect, and visual field pattern standard deviation. Biochemical parameters: BMI, total cholesterol, triglycerides, HDL, apolipoproteins A1, LDL, apolipoproteins B, ApoB/A1, lipoprotein a, neutrophils, monocytes, eosinophils, basophils, and lymphocytes.

The correlation analyses with the clinical and biochemical parameters showed that the PUFAs (LA and ALA) were positively correlated with CCT and superior and inferior pRNFL and negatively correlated with the visual field defects in the POAG patients (Figure 3). Saturated fatty acids and phenylalanine and their derivatives were positively associated with visual field defects and negatively associated with nasal pRNFL. The energy metabolite products (Pyruvic acid, Mannitol 1-phosphate, 2-Oxobutyric acid, and Methylsuccinic acid) were correlated with CCT, BCVA, and IOP. Lipid differentially abundant metabolites were correlated with HDL, ApoA1, and lymphocytes. Phenylalanine and its derivatives’ differentially abundant metabolites were correlated with the BMI, triglyceride, ApoA1, mononuclear cells, Eos, and lymphocytes. The energy metabolite products’ differentially abundant metabolites were correlated with total cholesterol, triglyceride, ApoB and lymphocytes.

### 3.3. Oxylipin-Targeted Metabolomics Analysis (OTMs)

**Figure 4:**
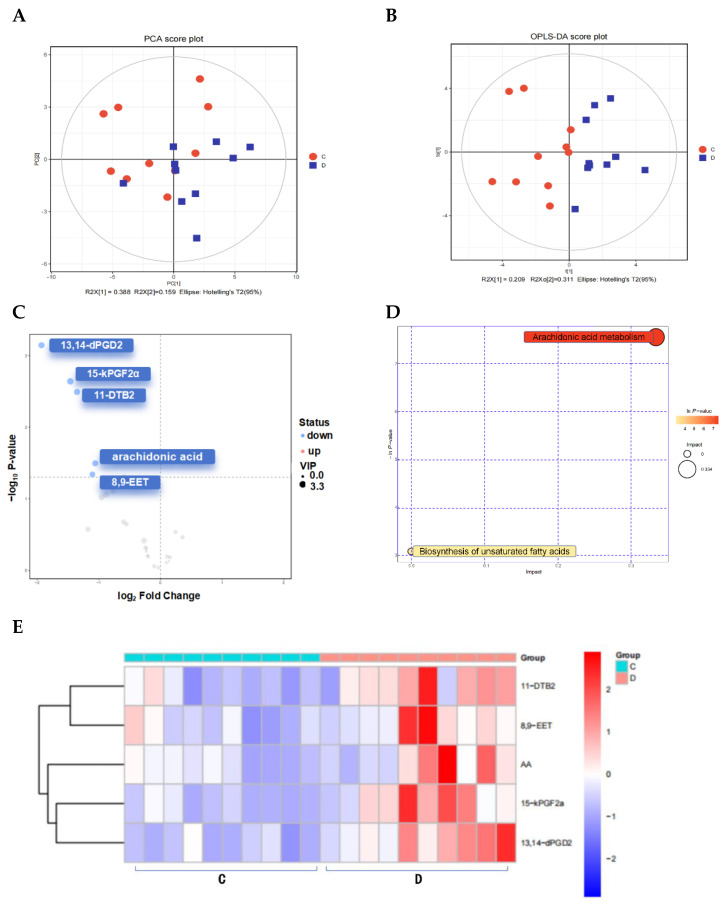

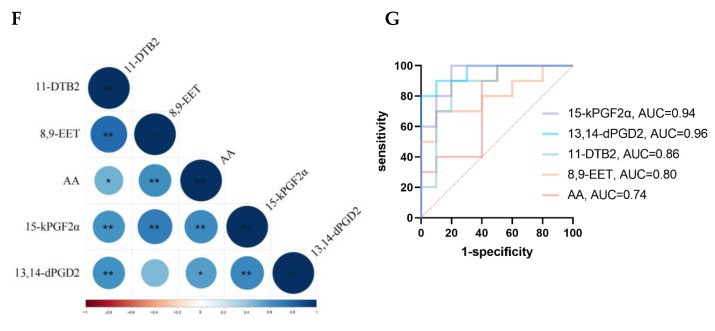
Oxylipin-targeted metabolomics analysis (OTMs). (**A**) PCA score plots and (**B**) OPLS-DA score plots of the two groups. (**C**) The volcano plot represents the screening of oxylipins. (**D**) Bubble plots indicate enrichment pathways. (**E**) Heatmap of oxylipins in the plasma between POAG and control. (**F**) Correlation heatmap of oxylipins, * *p*-value is less than 0.05, ** *p*-value is less than 0.01. (**G**) ROC curve analysis of oxylipins.

**Figure 5:**
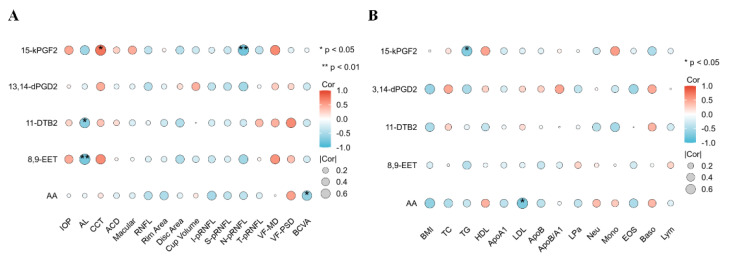
The correlation analysis of oxylipins with clinical and biochemical parameters. Correlation analysis between oxylipins and clinical (**A**) and biochemical (**B**) parameters.

The same samples of 10 POAG patients and 10 control subjects were used to determine the differentially abundant metabolites in the oxylipin-targeted metabolomics analysis. The PCA (Figure 4A) and the OPLS-DA (Figure 4B) model demonstrated the clustering of the samples within the same group and the differences between groups. The volcano plot identified 5 differentially abundant oxylipins out of 76 oxylipins in the plasma samples (Figure 4C), all of which were less abundant in the POAG group Table 5. 15-keto-Prostaglandin F2 alpha (15-kPGF2α) and 13,14-Dihydro-15-keto-prostaglandin D2 (13,14-dPGD2) are derived from prostaglandins (PGs). 11-Dehydro-thromboxane B2 (11-DTB2) is a product of thromboxaneA2 (TXA2), and eicosatrienoic acid (EET) generates 8,9-Epoxyeicosatrienoic acid (8,9-EET). Arachidonic acid is the upstream metabolite of them. The enrichment pathway analysis (Figure 4D) indicated that oxylipins were enriched in AA metabolism (impact = 0.33373, Raw *p* < 0.001). The Pearson correlation analysis demonstrated that 15-kPGF2α was positively correlated with CCT and negatively correlated with nasal pRNFL and TG in the POAG patients (Figure 5A,B). 11-DTB2 and 8,9-EET were negatively correlated with AL. AA was negatively correlated with BCVA and LDL.

### 3.4. UTMs Combine with OTMs

Based on KEGG metabolic pathway mapping, correlating UTM and OTM enrichment pathways, and the immunoinflammatory pathway with AA as the core, PUFAs upstream and oxylipins downstream was found to be the most characteristic pathway in plasma metabolic of POAG patients.

## 4. Discussion

Results from this study revealed that (1) the untargeted metabolomics analysis identified 33 differentially abundant metabolites, mainly lipids, phenylalanine and derivatives, and energy products; (2) the enrichment pathway analysis identified the metabolism of LA, ALA, phenylalanine, and TCA cycles; (3) 33 DEMs were correlated to clinical and biochemical parameters; and (4) the targeted metabolomics analysis identified 15-kPGF2α, 13,14-dPGD2, 11-DTB2, 8,9-EET, and AA as the differentially abundant oxylipins, which were enriched in the AA metabolism pathway. Collectively, this study delineated the metabolic profile in the plasma samples of the POAG patients.

Metabolic changes in lipid metabolism in POAG plasma include a decrease in PUFAs, corticosterone, and PC (16:0/16:0) and an increase in saturated fatty acids, sphingosine, and oxidized lipids. Genome-wide association studies have suggested an association of lipid-related genes with POAG [14]. In this study, the identified lipid differentially abundant metabolites included ALA and LA (Table 3), which belonged to ω-3 PUFAs and ω-6 PUFAs, respectively. They are the essential fatty acids needed to be obtained via food intake [20], and they are involved in cellular and mitochondrial membranes, regulating inflammation, and resisting oxidative stress damage [21]. The decrease in PUFAs observed in this study is consistent with the previous studies in elderly POAG patients [22] and POAG erythrocyte membranes [23]. The enrichment pathway analysis demonstrates that PUFAs are associated with AA metabolism, consistent with a previous targeted lipidomic analysis on aqueous humor of POAG patients [24,25]. The correlation analysis showed that the PUFAs are positively correlated with superior–inferior pRNFL thickness and negatively correlated with visual field defects in POAG patients (Figure 3), indicating that the PUFAs could be related to POAG development. Acar et al. [23] also found a linear correlation between the downstream metabolites of PUFAs in the plasma of POAG patients and visual field defects. PUFAs are abundantly distributed in the outer segments of retinal photoreceptor cells. A deficiency in PUFAs may lead to reduced membrane fluidity and diminished signal transduction capability, exacerbating local oxidative stress, all of which could be factors affecting the visual field [26]. PUFAs are essential components of mitochondrial membranes, critical for maintaining the process of oxidative phosphorylation [27]. Studies have indicated that a deficiency in PUFAs is a significant contributor to mitochondrial dysfunction [28]. Mitochondrial dysfunction produces a large amount of ROS and oxygen free radicals, causing oxidative stress damage, which is one of the important pathogenesis mechanisms of glaucoma [29].

Phenylalanine and its derivatives were found as another category of differentially abundant metabolites in this study; phenylalanine, acetophenone, phenylethylamine, and 4-hydroxybenzaldehyde were more abundance, whereas benzoic acid and phloroglucinol were less abundant, which is in contrast to previous findings that reduced phenylalanine and benzoic acid in both plasma and aqueous humor of POAG patients [9]. Phenylalanine is an amino acid and has a role in regulating inflammation [30]. Phenylalanine is turned into benzoic acid via β-oxidation [31], which has been reported to reduce inflammation by inhibiting microglia activation [9,32]. The correlation analysis showed that phenylalanine and its derivatives were negatively correlated with nasal pRNFL and positively correlated with visual field defects in the POAG patients (Figure 3). Interestingly, changes in phenylalanine and AA were also observed in a rat model of Alzheimer’s disease, implying that these two metabolites may participate in different neurodegenerative diseases. Nevertheless, further investigations are needed to validate this postulation [33].

Increased pyruvate and lactate but decreased citrate were found in this study. Pyruvate is generated from glucose by glycolysis, which further breaks down into lactate or acetyl CoA in the TCA cycle in the mitochondria [34], which was found in both plasma [7,17] and aqueous humor [35] of POAG patients and the DBA/2J glaucoma mouse model [36,37]. Impaired mitochondrial energy metabolism has been proposed to be related to POAG [18,19]. Mitochondria enrich metabolically active cells, such as cardiomyocytes and ganglion cells. In glaucoma patients, the RNFL is thinned, and large amounts of mitochondria are lost, causing impaired energy metabolism [29]. The injured mitochondria can produce reactive oxygen species and oxygen radicals [29], which can induce the oxidation of PUFAs.

Untargeted metabolomics analysis revealed the differentially abundant metabolites in the plasma samples of the POAG patients, including the lipid metabolites (LA and ALA). As PUFAs contain unsaturated bonds and are of short half-life and highly susceptible to oxidation to oxylipins, we further conducted the oxylipins-targeted metabolomics analysis to explore the relationship between oxylipins and POAG.

The oxylipins-targeted metabolomics analysis identified five oxylipins (15-kPGF2α, 13,14-dPGD2, 11-DTB2, 8,9-EET, and AA) less abundance in the POAG patients, and they are all enriched in the AA metabolism pathway.

15-kPGF2α and 13,14-dPGD2 belong to the prostaglandin-like oxylipins. 15-kPGF2α, a major stable oxidized product of PGF2α that reflects PGF2α biosynthesis and binds to the receptor FP [38,39]. 15-kPGF2α is considered to be a biomarker of COX-mediated inflammation, as well as assessing the level of oxidative stress [40]. Regrettably, research on 15-kPGF2α is limited, and the pathophysiological mechanism still needs further research.

13,14-dPGD2, also known as 13,14-D-15-kPGD2, is rich in the central nervous system and regulates inflammation by binding to the G protein-coupled receptors DP1 and DP2 on the cell membrane [41]. It has been reported that DP1 receptors play an anti-inflammatory role by inhibiting the JAK2-STAT1 axis of macrophages [42], while DP2 receptors are primarily involved in pro-inflammatory processes [43].

11-DTB2, a downstream metabolite of TXA2, has strong vasoconstrictor and platelet aggregation functions [44,45]. AA produces 11-DTB2, 15-kPGF2α, and 13,14-dPGD2 by the COX enzyme. The COX enzymes were highly distributed in the non-pigmented epithelium of the ciliary body, which produced aqueous humor. Moreover, COX-2 is lost in the patients with the end stage of POAG [46], indicating that the decreased AA and its downstream oxylipins may be related to the impairment in the COX enzyme system.

8,9-EET is mainly produced by vascular endothelial cells via CYP450 enzymes and has anti-inflammatory, anti-hypertensive, and cardioprotective effects [47,48]. 8,9-EET inhibits TNF-α-induced NF-κB-dependent transcription and VCAM-1 expression [49].

**Figure 6:**
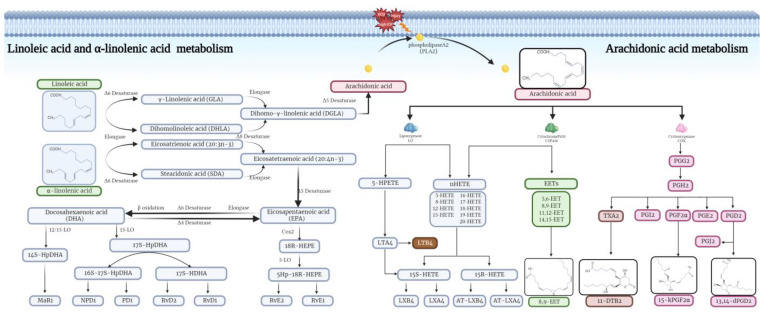
PUFAs (LA and ALA) generate AA, which binds to phospholipids inside the cell membrane. When the cell membrane is stimulated by external signals (high IOP, injury, or oxidative stress), phospholipase A2(PLA2) is activated, which hydrolyzes stable AA to the free state. COX, LOX, and CYP450 catalyze free AA to produce pro-inflammatory metabolites such as prostaglandins, thromboxanes, and leukotrienes and anti-inflammatory metabolites such as protectins, resolvins, and lipoxins, which co-regulate the immune-inflammatory response.

**Figure 7:**
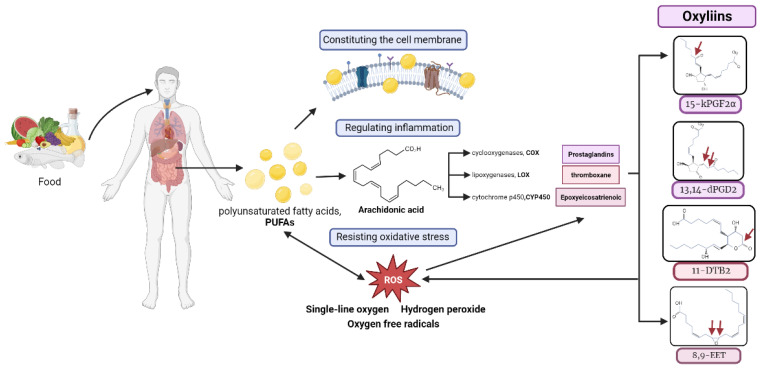
PUFAs, which are exclusively acquired via the diet, play a pivotal role in forming cellular membranes and modulating inflammatory and antioxidant responses. Reactive oxygen species (ROS) can oxidize PUFAs and AA to produce oxylipins. The resultant oxylipins exacerbate oxidative stress, underlining their potential impact on cellular homeostasis. Red arrows represent each oxylipin oxidized site.

The PUFAs (LA and ALA) generate AA [45], which binds to phospholipids inside the cell membrane. When the cell membrane is stimulated by external signals (high IOP, injury, or oxidative stress), phospholipase A2 is activated, which hydrolyzes stable AA to the free state. COX, LOX, and CYP450 catalyze free AA to produce pro-inflammatory metabolites such as prostaglandins, thromboxanes, and leukotrienes and anti-inflammatory metabolites, such as protectins, resolvins, and lipoxins [21,45,50,51]. Via the combination of untargeted metabolomics analysis with oxylipins-targeted metabolomics analysis, we found that the alterations in inflammatory pathways with AA as core, together with the upstream PUFAs and downstream oxylipins, suggests that the lipid metabolism change-mediated alterations in the AA metabolism pathway may be involved in POAG (Figure 6). The AA pathway is a recognized inflammatory cascade [45]. Oxylipins such as 9-HPODE and 15-kPGF2α, which are associated with oxidative stress levels [52] and serve as inflammation markers [38], respectively, point to oxidative stress and inflammation as potential promising directions for future research (Figure 7).

In this study involving 20 patients with POAG, nine patients were treated with prostaglandin analogs, specifically latanoprost or tafluprost. It is necessary to analyze the effects of drugs on metabolites. Multivariate linear regression analysis was conducted to evaluate the effects of drugs on metabolites, with the results indicating no significant differences. Additionally, Raber et al. [53] assessed the steady-state plasma concentration of 0.005% latanoprost eye drops in glaucoma patients, finding no significant clinical adverse events. The concentration curve for latanoprost revealed a peak concentration of approximately 20 pg/mL (1 pg = 0.001 μg), with complete metabolism occurring within 30 min. Furthermore, PCA and OPLS-DA models (Figure 1) suggested significant metabolomic differences between the POAG group and the control group in plasma, consistent with previous findings by Tang et al. [9]. Therefore, the impact of low-dose eye drops on plasma metabolites is very limited.

There are several limitations in this study. The metabolomics profiles of the patients were determined once only at the time of sample collection. As POAG is a chronic disease, the metabolomics profiles of the patients should be followed up in order to determine the metabolite changes along the disease progression.

The advancement of metabolomics is attributed to the development of equipment and the refinement of analytical methods, gradually integrating with artificial intelligence to efficiently identify biomarkers. Based on multi-omics analysis, it enables a comprehensive exploration of disease mechanisms. Its application in large-scale population screening, early clinical diagnosis, precision treatment, and prognosis assessment represents one of the most promising applications of metabolomics in medicine.

In summary, this study, based on the combination of the untargeted metabolomics analysis with the oxylipins-targeted metabolomics analysis, identified the differentially abundant metabolites and enriched metabolites pathways in the plasma samples of the POAG patients upon IOP lowering medication treatment. The results show that high IOP may not be the only detrimental factor for optic nerve cell damage in this group of POAG patients. The AA pathway and oxylipins could be involved in POAG. Specific roles played by each metabolite in POAG warrant further investigations.

## Figures and Tables

**Table 1:** Glaucoma medications used in the study subjects.

Patients	Medications	Duration	Severity
POAG-1	Brinzolamide and Timolol, Carteolol	5 months	moderate
POAG-2	Brinzolamide and Timolol, Brimonidine Tartrate	4 months	moderate
POAG-3	Brinzolamide and Timolol, Tafluprost, Latanoprost	2 years 2 months	severe
POAG-4	Brinzolamide and Timolol, Tafluprost	8 months	severe
POAG-5	Brinzolamide, Latanoprost	5 months	severe
POAG-6	Brinzolamide and Timolol	1 year 9 months	severe
POAG-7	Tafluprost, Brimonidine Tartrate	1 year 6 months	moderate
POAG-8	Brimonidine Tartrate	9 months	severe
POAG-9	Brinzolamide, Tafluprost	6 months	severe
POAG-10	Brinzolamide and Timolol, Brimonidine Tartrate	3 months	severe
POAG-11	Brinzolamide and Timolol	6 months	severe
POAG-12	Tafluprost, Carteolol	9 months	severe
POAG-13	Latanoprost, Brimonidine Tartrate	1 month	severe
POAG-14	Brinzolamide, Timolol, Tafluprost	7 days	moderate
POAG-15	Brinzolamide and Timolol	8 months	severe
POAG-16	Brinzolamide and Timolol, Brimonidine Tartrate	1 months	severe
POAG-17	Brinzolamide and Timolol	5 months	severe
POAG-18	Brinzolamide and Timolol, Tafluprost	3 months	severe
POAG-19	Brinzolamide and Timolol	1 year 7 months	severe
POAG-20	Brinzolamide and Timolol, Brimonidine Tartrate	7 days	mild

Mild glaucoma (mean deviation (MD) ≥ −6 dB), moderate glaucoma (−6.01 to −12.00 dB), and severe glaucoma (MD <−12 dB).

**Table 2:** Demographics and clinical and biochemical parameters of the POAG and healthy control subjects.

	POAG (*n* = 20)	Controls (*n* = 20)	*p*
Age (years)	68.5 ± 7.41	66.85 ± 8.33	0.512 ^a^
Sex (male/female)	14/6	11/9	0.330 ^b^
Smoking (yes/no)	13/7	10/10	0.340 ^b^
BMI (kg/m^2^)	24.20 ± 3.06	22.84 ± 3.24	0.192 ^a^
SBP (mmHg)	138.2 ± 16.09	133.25 ± 11.47	0.270 ^a^
DBP (mmHg)	79.90 ± 10.06	81.30 ± 7.84	0.627 ^a^
BCVA (logMAR)	0.50 ± 0.30	0.69 ± 0.63	0.119 ^a^
IOP (mmHg)	14.98 ± 3.52	14.13 ± 3.05	0.420 ^a^
AL (mm)	23.23 ± 0.80	23.63 ± 0.70	0.100 ^a^
CCT (µm)	549.12 ± 42.98	532.85 ± 30.56	0.126 ^c^
ACD (mm)	3.20 ± 0.25	3.15 ± 0.42	0.692 ^a^
C/D	0.9(0.8, 0.9)	0.3 (0.3, 0.4)	<0.001 ^c^
VF (MD)	20.31 ± 7.76	-	-
VF (PSD)	7.68 ± 1.84	-	-
TC (mmol/L)	5.45 ± 1.11	4.77 ± 0.76	0.031 ^a^
TG (mmol/L)	1.74 ± 0.83	1.34 ± 0.59	0.123 ^c^
HDL (mmol/L)	1.59 ± 0.66	1.35 ± 0.40	0.159 ^a^
LDL (mmol/L)	3.23 ± 1.28	2.91 ± 0.78	0.339 ^a^
LP(α) (mg/L)	137.55 ± 108.77	138.50 ± 124.49	0.787 ^c^
Apo A-1 (g/L)	1.31 ± 0.22	1.26 ± 0.21	1.000 ^c^
Apo B (g/L)	0.93 ± 0.26	0.85 ± 0.14	0.249 ^a^
Apo B/A-1	0.71 ± 0.18	0.70 ± 0.17	0.802 ^a^
Glucose (mmol/L)	6.79 ± 1.76	6.33 ± 1.41	0.344 ^c^
WBC (×10^9^/L)	7.84 ± 2.25	7.03 ± 2.09	0.267 ^a^
Neu (×10^9^/L)	4.76 ± 1.71	4.25 ± 1.56	0.345 ^a^
Lym (×10^9^/L)	2.25 ± 0.65	2.07 ± 0.77	0.306 ^c^
Mono (×10^9^/L)	0.66 ± 0.24	0.49 ± 0.15	0.017 ^c^
Eos (×10^9^/L)	0.18 ± 0.20	0.16 ± 0.15	0.644 ^c^
Baso (×10^9^/L)	0.12 ± 0.15	0.06 ± 0.04	0.270 ^c^

BMI, body mass index; SBP, systolic blood pressure; DBP, diastolic blood pressure; BCVA, best corrected visual acuity; IOP, intraocular pressure; AL, axial length; CCT, central corneal thickness; ACD, anterior chamber depth; C/D, cup/disc ratio; MD, visual field main defect; PSD, visual field pattern standard deviation; TC, total cholesterol; TG, triglyceride; HDL, high-density lipoprotein; LDL, low-density lipoprotein; LP(α), lipoproteinsα; Apo A-1, apolipoproteins A-1; Apo B, apolipoproteins B; WBC, white blood cell; Neu, neutrophils; Lym, lymphocytes; Mono, mononuclear cells; Eos, eosinophils; Baso, basophilic granulocytes; Continuous variables were presented as mean ± SD according to the normality of the data. Categorical variables were presented as proportions. Statistical test: ^a^ Student’s *t*-test. ^b^ Fisher’s exact test. ^c^ Mann–Whitney U test.

**Table 3:** Differentially expressed metabolites in the plasma samples of the POAG group compared to the healthy control group.

Metabolites	VIP	FC	*p*	AUC	
Acetylcholine	1.4	38.1	0.0345	0.73	**↑**
Cotinine	1.35	2.66	0.0283	0.77	**↑**
9-HpODE	1.35	2.08	0.011	0.7	**↑**
Pyruvic acid	1.29	1.55	0.0013	0.8	**↑**
Sphingosine	1.09	1.43	0.0248	0.68	**↑**
Phytosphingosine	1.07	1.41	0.0306	0.68	**↑**
Hexadecanamide	1.43	1.34	0.0116	0.76	**↑**
4-Hydroxybenzaldehyde	2.13	1.29	<0.001	0.84	**↑**
Phenethylamine	2.6	1.28	<0.001	0.91	**↑**
L-Phenylalanine	1.22	1.27	<0.001	0.86	**↑**
L-Lactic acid	1.36	1.23	0.0084	0.71	**↑**
4-Phenylbutyric acid	2.1	1.18	<0.001	0.85	**↑**
Acetophenone	2.43	1.16	<0.001	0.91	**↑**
Palmitic acid	1.16	1.06	0.0217	0.73	**↑**
Corticosterone	−1.22	0.91	0.007	0.79	↓
Citric acid	−1.15	0.89	0.0356	0.72	↓
Cytidine	−1.89	0.89	<0.001	0.83	↓
Tretinoin	−1.82	0.89	<0.001	0.81	↓
4-Oxoproline	−1.07	0.87	0.0245	0.69	↓
Lauric acid	−1.64	0.86	0.0042	0.78	↓
4-Toluic acid	−2.44	0.84	<0.001	0.95	↓
Benzoic acid	−2.62	0.8	<0.001	0.98	↓
Methylsuccinic acid	−1.05	0.77	0.0399	0.66	↓
Levulinic acid	−2.53	0.76	<0.001	0.98	↓
16-Hydroxyhexadecanoic acid	−1.32	0.72	0.0348	0.72	↓
Mannitol 1-phosphate	−1.88	0.71	<0.001	0.81	↓
PC(16:0/16:0)	−1.42	0.69	0.009	0.73	↓
L-Arginine	−1.51	0.67	0.0405	0.7	↓
Linoleic acid	−1.5	0.63	0.0056	0.74	↓
trans, trans-Muconic acid	−2.33	0.62	<0.001	0.96	↓
Phloroglucinol	−1.05	0.54	0.0499	0.69	↓
alpha-Linolenic acid	−1.19	0.54	0.015	0.72	↓
2-Oxobutyric acid	−1.51	0.51	0.0215	0.86	↓

VIP, variable importance projection; FC, fold change; AUC, area under the ROC curve. In total, 33 differentially abundant metabolites were identified with |VIP| > 1 and *p* < 0.05. ↑ represents an increase compared to the control; ↓ represents a decrease compared to the control.

**Table 4:** The pathway enrichment analysis.

Pathway	Total	Hits	Raw *p*	−ln(*p*)	Impact
**Linoleic acid metabolism**	5	2	0.0022344	6.1038	1
**Phenylalanine metabolism**	10	2	0.0095891	4.6471	0.59524
**alpha-Linolenic acid metabolism**	13	2	0.016156	4.1255	0.33333
**Citrate cycle (TCA cycle)**	20	2	0.045787	3.0838	0.13672

Pathway, metabolic pathway name; Total, number of metabolites in the pathway; Hits, number of differential metabolite hits in the pathway; Raw *p*, *p*-value for metabolic pathway enrichment analysis; Impact, impact value for metabolic pathway topology analysis.

**Table 5:** OTMs for oxylipins in plasma of POAG and control subjects.

Metabolites	VIP	FC	*p* Value	AUC	
15-kPGF2α	−1.95	0.36	0.0023	0.940	↓
13,14-dPGD2	−1.60	0.26	0.0007	0.960	↓
11-DTB2	−1.75	0.39	0.0032	0.860	↓
8,9-EET	−1.56	0.48	0.0319	0.800	↓
AA	−1.35	0.47	0.0456	0.740	↓

OTMs identified 5 oxylipins with criteria of |VIP| > 1 and *p* < 0.05. ↓ represents a decrease compared to the control.

## Data Availability

The original contributions presented in the study are included in the article/supplementary material, further inquiries can be directed to the corresponding author/s.
